# Nurses’ and midwives’ experiences of providing group antenatal and postnatal care at 18 health centers in Rwanda: A mixed methods study

**DOI:** 10.1371/journal.pone.0219471

**Published:** 2019-07-11

**Authors:** Tiffany Lundeen, Sabine Musange, Hana Azman, David Nzeyimana, Nathalie Murindahabi, Elizabeth Butrick, Dilys Walker

**Affiliations:** 1 University of California San Francisco, Institute of Global Health Sciences, San Francisco, California, United States of America; 2 University of Rwanda School of Public Health, Kigali, Rwanda; 3 University of California San Francisco, Department of Obstetrics, Gynecology, and Reproductive Sciences, San Francisco, California, United States of America; Liverpool School of Tropical Medicine, UNITED KINGDOM

## Abstract

**Background:**

The East Africa Preterm Birth Initiative-Rwanda began a cluster randomized controlled trial of group antenatal care (ANC) and postnatal care (PNC) in Rwanda in 2017. That trial will report its primary outcome, gestational length at birth, after data collection concludes in 2019. This nested study includes providers of ANC and/or PNC at the 18 health centers randomized to provide the group model of ANC/PNC and the 18 health centers randomized to continue providing ANC/PNC in the traditional, individual visit model. The objective of this study is to understand the experiences of providers of group ANC/PNC and compare their job satisfaction and perceived stress with individual ANC/PNC providers.

**Methods:**

We collected both quantitative and qualitative data from providers (nurses and midwives) who were recruited by health center directors to participate as group ANC and PNC facilitators at intervention sites and from a similar number of providers of standard ANC and PNC at control sites. Quantitative data was collected with questionnaires administered at baseline and approximately 9 months later (follow up). Qualitative data was collected in 3 focus groups of group ANC/PNC providers conducted one year after group care began.

**Results:**

Eighty-six percent of nurses and midwives surveyed who implemented group ANC and PNC reported that they prefer group care to the traditional individual model of ANC and PNC. Perceived stress levels and job satisfaction results were similar between groups. Mixed focus group discussions among both nurses and midwives experienced in group ANC and PNC suggest that the group model of care has advantages for both service beneficiaries and providers. When providers described implementation challenges, their peers in the focus groups offered them suggestions to cope and improve service delivery.

**Discussion:**

These results are consistent with studies of providers of group ANC and PNC in other LMIC contexts with respect to the perceived benefits of group care. This study adds new insights into the ways peer providers can help one another solve implementation problems. When given the opportunity to meet as a group, these study participants offered one another peer support and shared knowledge about best practices for successful implementation of group ANC/PNC.

This trial is registered at clinicaltrials.gov as NCT03154177.

## Introduction

### Group antenatal and postnatal care

Group antenatal care is an alternative to traditional, individual antenatal care (ANC) visits. In the group ANC service delivery model, 8–12 women with similar estimated due dates are organized into groups that meet at regular intervals during pregnancy to access all routine ANC services with the assistance of at least one ANC provider. Group ANC replaces individual ANC visits for eligible women, and can be an adjunct opportunity for women who may need some individual visits for high-risk conditions.[1] Because postnatal care is a key component of family-centered care during the childbearing year, group postnatal care (PNC) has also been implemented by some programs; group ANC participants may return for group care at about 6 weeks after birth or in some cases at regular intervals during the child’s first year of life.[2–4]

In addition to the participating beneficiaries, i.e. pregnant women and their partners or parents of the newborn, group ANC visits are led by one or more “facilitators.” Group care programs generally rely on two facilitators to be present at each group visit; one facilitator is a licensed clinician such as a nurse, midwife, or physician, while the second facilitator may be either a clinician, community health worker, or other health specialist (for example, a lactation or nutrition counselor, social worker, or student clinician). The facilitators are responsible for organizing the group visit and accomplish health assessments and group discussion of relevant topics with as much beneficiary participation as possible.

In group ANC and PNC care, education and support are provided in a group. Health assessment takes place in the group space and women participate in their own physical assessment; women who read and write can be coached to measure and record their own weight and blood pressure, using an electronic blood pressure cuff. Group care facilitation promotes participatory learning and sharing of experiences; evidence suggests participants have increased knowledge and are more satisfied.[3,5]

Facilitation is a skill that requires training, practice, and feedback, as it is quite different than a didactic method that providers may use to instruct beneficiaries in antenatal “classes.” The importance of excellent facilitation during group ANC visits has been documented in the literature. A 2013 study examined the associations between fidelity to effective group ANC process (including effective facilitation and group member engagement), fidelity to planned group ANC content, and the health outcomes of group ANC participants at two urban health facilities in the United States.[6] In this study, group ANC participants were, in terms of demographic descriptors, those at the highest risk of poor perinatal outcomes in the United States. Facilitation and engagement during group care visits, or process fidelity, was associated with a significantly decreased risk of preterm birth and decreased newborn admissions to the intensive care unit. Content fidelity was significantly related to decreased newborn intensive care unit admission, but not to preterm birth. Other authors have emphasized that even though providers with some degree of social status facilitate the sessions, the group ANC process must be non-hierarchical so that women can draw on their own strengths and increase their self-efficacy as individuals and as a community of mothers.[7–8]

Published reports of group ANC implementation are available from at least 16 countries. Most of these refer to group ANC’s effects on health outcomes and women’s experience of care. Seven reports of qualitative data from group ANC facilitators are available: one from the United Kingdom,[8] one from Malawi and Tanzania,[9] one from Australia,[10] 2 from Canada,[11–12] and 2 from the United States.[13–14] The United Kingdom study reported that 67% of providers who implemented group ANC (12 midwives in this study) felt that it was an improvement over standard ANC and 33% of providers rated it as somewhat better than standard ANC.[8]

### Group ANC/PNC in Rwanda

In 2017, the East Africa Preterm Birth Initiative-Rwanda implemented group antenatal care and postnatal care in 18 health centers in Rwanda, in the context of a cluster randomized controlled trial. The primary outcome of the trial is gestational length and data collection is ongoing. A group of Rwandan stakeholders, including a nurse, a midwife, physicians, officials from the Ministry of Health, and public health researchers, planned for this implementation; their process is described elsewhere.[2] This group of stakeholders made two context-specific decisions. First, they decided that after an initial, one-on-one ANC visit pregnant women would be invited to attend three group ANC visits during pregnancy and one group PNC visit at approximately 6 weeks after birth. Second, the stakeholders decided that the optimal combination of two facilitators at each group visit, within the unique context of the Rwanda public health system, would be one ANC or PNC provider and one community health worker. The Rwanda health care system is a pyramidal structure of 8 national referral hospitals, 4 provincial hospitals, 36 district hospitals in 30 districts, 504 health centers, and 670 health posts, and includes 58,286 community health workers (CHWs) at the village level.[15] Health centers are staffed only by nurses and midwives and must provide a package of primary services. Staff are rotated as needed across services. In addition to other services universally available at the health center level, nurses and midwives provide antenatal, intrapartum, and postnatal services. The 2014–2015 Rwanda Demographic and Health Survey reported that nurses provide 93.7% of antenatal care and midwives provide 0.6% of antenatal care; the remainder is provided by physicians at referral hospitals for complex cases.[16] Some community health workers have special interest and training in maternal and child health surveillance.

After an in-depth orientation to the trial objectives and methods, directors of the 18 health centers randomized to group care for this trial each selected 3 providers to be trained and act as group care facilitators. These selected provider-participants were either nurses or midwives, were frequently assigned to ANC or PNC services at their respective health centers, and were required to fulfil this new group care facilitator role as part of their regular job duties. Ministry of Health officials preferred that health center directors choose which providers to include, relying on their facility-specific staffing knowledge. A distinct community leader, the Supervisor of Community Health Workers (CHWs) associated with each of the 18 group care study sites, selected 12 CHWs to be trained and act as group care co-facilitators. A team of 6 Rwandan group care Master Trainers prepared those 72 providers and 216 community health workers to act as co-facilitators during group visits, training them in small mixed groups. Master Trainers focused on building facilitation skills during a three-day orientation to group care, and then reinforced these skills during supportive visits made to each health center every other month for 12 months. A group ANC/PNC manual, created by the Rwandan stakeholder group, was given to each health center, and providers were encouraged to study it and use it as a reference during every group visit. Study staff invited trained providers to reach out at any time with questions or concerns about group care implementation.

The parent study conducted by the East Africa Preterm Birth Intiative-Rwanda includes the analysis of multiple outcomes, including gestational length, ANC coverage and women’s experiences in group ANC/PNC; those results will not be reported in this article. This nested study seeks to understand the experiences of Rwandan nurses and midwives one year after the implementation of group ANC and PNC in 18 health centers in order to inform policy makers as they consider the feasibility of scaling up this alternative model of care.

## Methods

We collected both quantitative and qualitative data from nurses and midwives who were recruited by facility directors to participate as facilitators for the East Africa Preterm Birth initiative trial of Group ANC and PNC in Rwanda.

### Study setting

Five of 30 Rwandan districts were selected for this trial in collaboration with the Rwanda Ministry of Health. Within those 5 districts, 55 health centers were assessed with a standardized tool for number of providers, ANC volume, suitable space for group care, services, and equipment. Health centers that reported, in this facility assessment, that they allocate at least 2 providers to ANC services on any day that ANC is offered were selected for this trial, for a total of 36 health centers. These 36 health centers were pair-matched and then randomized to either continue individual ANC and PNC or switch to group ANC and PNC. The 18 health centers randomized to implement group ANC and PNC are located in rural, peri-urban, and urban locations.

### Participant recruitment and data collection: Questionnaires

Providers trained to be group ANC/PNC facilitators at the 18 health centers randomized to group care were invited to participate in a longitudinal survey, with a questionnaire administered at baseline (training), and at approximately 9 months after implementation. At each of the 18 health centers randomized to continue traditional, one-on-one ANC/PNC visits (control), the providers most frequently allocated to ANC/PNC were invited to participate in the same longitudinal survey (average 3 providers); these were also selected by facility directors based on facility-specific staffing patterns. Questionnaires were also administered to control providers at similar time points. Providers did not receive any incentive to participate in this study.

The survey instruments include questions about education, license/title, and years of work experience in order to describe the cohort and look for associations with model preference over time. To assess job satisfaction, we asked, “Thinking specifically about your work in ANC and/or PNC services, how would you rate your current job satisfaction on a scale from 1–5, with 1 representing extremely dissatisfied and 5 representing extremely satisfied?” We used the Perceived Stress Scale to measure stress.[17] Questions and answers appeared in the survey in both English and French; provider training at secondary and post-secondary schools is in one of these languages, and the written form of Kinyarwanda is not standardized. These questionnaires appear in S1 Doc.

Questionnaires were self-administered by providers entering data on a tablet that syncs to an electronic data capture system.[18] If the tablet was not available, providers completed the questionnaires on paper and a data collector entered the data to the electronic data capture system. Questionnaires did not collect name or other personal identifiers; each provider participant was assigned a unique study identification code and this was used on all questionnaires, both at baseline and follow-up.

### Participant recruitment and data collection: Focus groups

Twelve months after implementation, we convened three focus groups for group ANC/PNC providers. Health center directors at the 18 intervention sites selected for these focus groups the providers who had been most actively involved in group care provision over the past year. The selected providers were granted “time off” by their supervisors to attend the focus group discussions, which were held in a geographically central location.

These 3 focus groups were convened at a hotel, one group per day for 3 consecutive days, with an average of 10 provider participants in each group. They received reimbursement for their travel expenses but did not receive an additional incentive. Each focus group discussion (FGD) lasted 60 minutes, was conducted in Kinyarwanda, and was facilitated by one of the group ANC/PNC Master Trainers. The focus group facilitator followed a guide with 4 open-ended questions, to allow the nurses and midwives to freely verbalize their ideas and concerns about group ANC/PNC (S2 Doc). Focus group discussions were audio recorded and each participant was assigned a number; facilitators referred to these numbers when calling on participants during the discussion. Also present during the FGDs was the Principal Investigator [SM] and the group ANC/PNC technical advisor [TL], who asked the providers to speak freely because their inputs are vital to improving the program, reassured them about confidentiality, and emphasized that there is no personal or professional hierarchy among providers and researchers.

### Analysis and interpretation

Quantitative data from the questionnaires were analyzed using simple descriptive statistics. We used the five steps of the Framework Method to analyze 3 FGDs: familiarization, identify a thematic framework, indexing, charting, and mapping and interpretation.[19] Audio recordings of FGDs were transcribed verbatim and translated from Kinyarwanda to English; this translation was reviewed by one of the authors, who is fluent in Kinyarwanda, for accuracy (SM). Transcripts were not returned to the participants for comment or correction. Next, one author coded one full FGD transcript, assigning 1–3 codes to every phrase, sentence, and/or paragraph (TL). Two authors then met to discuss the codes, group them, and organize them into a working analytical framework (EB and TL). Sixty-three codes were grouped into 12 new codes, in 6 main categories, plus an “other” category. The codes for this analytical framework were then applied to the two remaining FGD transcripts and the data was charted into a framework matrix. The authors generated themes by reviewing this matrix, keeping in mind our original research objectives while simultaneously looking for unexpected results. These codes and themes were reviewed independently by a third author (SM).

### Ethical considerations

Ethical approval for the survey and the focus group activities was granted by the Rwanda National Ethics Committee (No.0034/RNE/2017) and University of California, San Francisco Institutional Review Board (16–21177). Written informed consent was obtained from each participating provider prior to administering the baseline questionnaire. A separate written informed consent was obtained from providers participating in a FGD. Only participants who agreed to participate and signed the consent form were invited to complete the questionnaires or join the FGDs. Moderators obtained permission from FGD participants to record the conversation. Voluntary participation was ensured throughout the study. All information was kept confidential and personal identifiers were not recorded on audio and written files.

## Results

### Quantitative results

Fifty-nine providers at intervention sites and 46 providers at control sites completed questionnaires at both baseline and a follow-up time point ranging from 3 to 12 months after baseline. Over time, there was attrition among provider participants at both group ANC and traditional ANC study sites for various personal or professional reasons. For this reason, not all 72 providers originally trained in group care are represented in this sample. While 29 providers trained in group ANC/PNC participated in the 3 focus groups, only 26 of those completed both a baseline and follow-up questionnaire. The basic characteristics of group care providers, control site providers, and focus group participants were similar and are presented in Table 1. The average job satisfaction and Perceived Stress Scale scores at both time points were similar among all groups. Group care providers were asked in the follow-up questionnaire, “How do you prefer to provide antenatal and/or postnatal care—individual visits, group visits, or no preference?” Eighty-six percent preferred to provide group ANC and PNC instead of individual ANC and PNC, while 9% reported no preference and 5% preferred to deliver individual ANC and PNC. Because such a large majority preferred group ANC/PNC to individual ANC/PNC and the sample size is small, there were no clear associations between model preference and title, education, service preference, age, or years of ANC/PNC experience. Providers of group ANC/PNC reported that they had, on average, facilitated 26 group visits in the previous 9–12 months (minimum number of visits was 5, maximum number of visits was 130).

**Table 1:** Responses of provider participants who completed both baseline and follow-up questionnaires.

Characteristic	Group care providers at baseline	Control providers at baseline	Focus group participants at baseline	Group care providers at follow-up	Control providers at follow-up	Focus group participants at follow-up
Age: n	59	46	26			
Average years of age (range)	36 (24–51)	36 (25–53)	36 (24–51)			
Title: n	59	46	26			
Nurse: %	81	72	69			
Midwife: %	19	28	31			
Highest level of education: n	58*	46	26			
Secondary school: %	24	24	15			
Post-secondary vocational training: %	5	11	0			
University: %	71	65	85			
Years of work: n	53*	46	26			
In ANC and/or PNC: average (range)	5.8 (1–29)	6.7 (1–17)	5.8 (1–29)			
At the facility where you currently work: average (range)	7.0 (1–17)	7.0 (1–17)	6.6 (1–16)			
Preferred service area: n	56*	46	25*			
ANC: %	50	54	60			
Maternity/Labor & Delivery: %	29	22	40			
HIV: %	9	4	0			
PNC: %	2	0	0			
PMTCT: %	5	7	0			
Vaccinations: %	4	7	0			
Other: %	1	6	0			
Perceived Stress Scale (PSS): n	52*	46	24*	52*	46	24*
Average score (range)	13.1 (2–20)	12.9 (3–24)	11.6 (2–17)	11.4 (0–23)	10.3 (0–22)	10.9 (0–20)
Low stress score (0–13), %	52	50	63	72	65	79
Moderate stress score (14–27), %	48	50	37	28	35	21
Job satisfaction related to work in ANC/PNC, on a 1–5 scale (1 is lowest, 5 is highest satisfaction): number of responses	57*	46	26	57*	46	26
Average (range)	3.9 (1–5)	4.0 (1–5)	4.1 (1–5)	3.8 (1–5)	3.8 (1–5)	3.8 (1–5)
Dissatisfied: %	11	8.5	8	14	11	15
Neutral: %	12	8.5	4	9	13	8
Satisfied: %	77	83	88	77	76	77
Model preference: n				58*		26
No preference: %				9		8
Individual visits: %				5		11
Group visits: %				86		81

n = number of responses

*If the data item was missing from either the baseline or follow-up questionnaire, the participant was excluded from analysis for that question.

Dark grey shading: No data expected in these areas of table.

### Qualitative results

Themes and sub-themes that emerged from the FGDs are summarized in Table 2. The two over-arching themes are that 1) group ANC and PNC offer advantages to both mothers and providers compared to individual ANC and PNC, and 2) implementation challenges can be addressed by following best practices shared by provider-peers during these focus group discussions.

**Table 2:** Themes, sub-themes, and details of focus group discussions among Rwandan providers of group ANC and PNC.

Theme	Subtheme	Details
There are advantages to group care	Providers enjoy GANC; providers’ satisfaction with care is increased	More emotional connection to women in their care
		Increased pride in the quality of their work
		Mothers’ increased health-related knowledge results in increased health-seeking behaviors
	Women enjoy GANC; women’s engagement in ANC is increased	Women are free to express concerns, beliefs, and practices that they did not share before
		Women are “thirsty” for knowledge
		Women share information obtained in GANC with the community
Best practices to meet implementation challenges	Implementation challenges at the facility level--and solutions	Ideal group visit scheduling
		Ideal staffing strategy (especially allocation)
		Managing altered workload
	Obstacles to women’s access	Payment of Mutuelles
		Poor attendance at GPNC
		Long distance to health center

#### Theme 1: Advantages of group ANC/PNC

Providers report that they experience increased satisfaction with ANC and PNC in the group visit model, and they perceive that women are also more satisfied with group ANC and PNC.

*As for me*, *this group care program has pleased us very much; you can even learn of this fact through much excitement of the group members*. *For us who lead group care*, *we can see it*. *You can see that mothers are thirsty for knowing all those new things*. *When you discuss with them and when you are making conclusions together with them*, *you find the members happy*, *and most of them wish never to miss out*. *A woman says that she is happy to learn something new*.

(FG2 R4)

The most commonly stated sources of this satisfaction are freedom of expression, increased quality of care (including health education), and relationships among group members.

**Freedom of expression**

Several providers remarked that women participating in group care visits freely expressed their concerns and opinions and shared their personal experiences in a completely new way. In turn, providers felt free to share their time, friendship, and knowledge with mothers.

*In the past*, *pregnant women used to come and listen to a brief talk from the nurse*. *But today*, *they come and sit together with the nurse and share*. *They ask questions and get answers to them*. *In the past*, *the nurse could fail to get time to answer to their questions; so they could go back home without answers*. *Today*, *they are free to ask whatever they want; they feel at ease with the nurse; they behave like friends*.

(FG2 R2)

*I am very much satisfied [with group ANC/PNC]*. *I would say that the success results from freedom*. *When we have come together*, *we sit and talk freely with those mothers whom we serve*.

(FG3 R7)

This freedom of expression is possible within warm and trusting relationships and results in vulnerable and fruitful information-sharing.

*When we have held our discussions in group care*, *the clients feel free to talk because we trained them into keeping the secret*. *This free talk is due to the fact that no one hears outside there what she has said during discussion within the group care*. *Because of that she will be free to tell of her experience which allows us all to work together to find remedy to her problems*.

(FG1 R9)

**Increased quality of care**

The most prominent sub-theme was that pregnant women who participate in group ANC/PNC influence one another to adopt healthy behaviors by sharing their own real-life stories. This increase in health literacy was noted by providers as an advantage to both women and providers, who emphasized four main domains in which they had noticed changes in women’s behavior that were clinically significant. First, women learned from one another to recognize danger signs and and seek timely care:

*[A] woman helped other women in the group care to understand alarming signs of convulsion [that she had experienced herself]*. *They were shocked by their previous beliefs about convulsion which they qualified as a demon possession state*. *They made up their minds that they would go at once to the health center as soon as they see such signs as those of that very woman*.

(FG1 R uncertain)

Second, women ceased using traditional medicines during pregnancy, a common practice in Rwanda believed to protect against poor perinatal outcomes:

*For example a woman in my group said*, *“Before I joined this group*, *I was told that if a pregnant woman does no take traditional medications*, *her child may suffer from skin diseases and other diseases*. *But now I know that it was a lie*.*” They say this because other women [in the group] tell how they gave birth to many children without having taken the traditional medications*, *yet their children didn’t have any problem*. *It leads many to concluding never to use them*. *They were free to share any information and hence get rid of all kind of rumors related to the usage of those traditional medications*.

(FG2 R4)

Third, women changed their attitudes about postnatal family planning:

*“* … *a certain woman gave us [her testimony] when we were discussing about family planning in our group care*. *The group members had a lot of rumors about family planning*, *and she told us how soon after she had delivered her baby*, *she went and planned for her births*. *She related how she was successful in spacing her births by using the implant method of family planning*. *Her testimony built the understanding of her peers so much*.

(FG1 R3)

And fourth, more women were persuaded to give birth at a facility:

*We used to have a big number of women who deliver from their homes; even those who would deliver on their way to the health center would go back home at once without reaching the health center; today such cases have reduced*. *Women come and deliver from the health center; it is something interesting*.

(FG3 R1)

Providers recount that women are both “thirsty” for knowledge and eager to share what they have learned with others. Providers note that “the pregnant mothers share information and learn from one another the information; we do not deliver it to them” (FG 3 R7). Furthermore, women who participate in group ANC/PNC share their experiences and knowledge with others in the community:

*Something that touched my heart is the fact that many pregnant mothers responded positively to the invitation from their neighbors to join this program of consulting in groups*. *After some of their friends had gone to the health center and found out the services the program was providing*, *they encouraged others and the latter attended*.

(FG3 R5)

Some providers admitted that the structure of group care visits resulted in an increase in routine assessments, especially blood pressure:

*We didn’t use to test blood pressure*, *and the effect resulting thereof could take the lives of many women*. *This test is very important*. *[In the past] it was very possible [we did not check blood pressure] even until she gives birth*. *They [group care participants] can test that blood pressure themselves because they already know how to do it*. *When they have tested one another and found out that there is one who has a problem*, *they inform the nurse*, *and the nurse can verify and provide due assistance to the woman having the problem before the situation becomes worse*. *Things have become very easy*.

(FG 3 R5)

**Relationships**

*This Ibaruke Neza [group ANC/PNC] program which is carried out in the groups made me like my job*. *Why is that*? *Clients have lovely and friendly interactions with nurses*, *they feel at ease when talking with them*.

(FG1 R3)

Providers experienced the dissolution of hierarchies between themselves and their clients as satisfying and therapeutic.

*The group care program has brought the nurses closer to their clients*. *Before one could see a nurse as someone who is in a very high level*, *but today we can talk and laugh together in groups*. *We find that we have freedom; even when a woman has a problem she comes again and asks you*. *In fact*, *it is like a friendly relationship between the woman and the nurse*. *She considers you as a sister rather than a health center employee*.

(FG2 R7)

#### Theme 2: Best practices to overcome implementation challenges

An interesting process result of these FGDs was that once an implementation challenge was mentioned by a participant, peers in the same FGD offered their own suggestions for best practices to overcome them. The process for each FGD was similar to that of a group ANC or PNC visit, with a free exchange of stories and information among peers. We have divided these implementation challenges into 2 main sub-themes: implementation mistakes and solutions at the facility level; and obstacles to women’s access.

*Implementation challenges at the facility level*--*and solutions*

By far, the most commonly cited problem is a shortage of provider staff at health centers. When there are few staff available to cover all the basic service units at the health center, it is common for the health center director to allocate a single provider to several services at once—such as maternity, ANC, and family planning. Stress and chaos can result:

*It happens sometimes for a service provider to feel stressed due to the fact that we work in more than one service; we get a challenge of failing to render an adequate service because the group care activities require you sit in one place--to stay there*. *It happens that you may be urgently needed in a different service; you are thus obliged to excuse yourself for an absence of little while to provide the services called for*, *and this becomes disturbance*. *You may be working from maternity and find yourself at the same time in post natal group care of those very mothers; so you feel puzzled*. *It is due to the little number of workers whereby we are bound to combine services because they are more than the number of workers available*.

(FG3 R6)

Peers in the FGDs suggested several approaches to this problem. One solution is that staff should give special consideration to group care and creatively cover all services over the course of a day:

*This issue of the lack of enough personel is almost everywhere in the health centers*. *I would advise that the person handling group care would be left alone to carry out that task first*, *and assign duty in two*, *even three services to other people who are not handling group care*. *Thus*, *this person handling groups will go to assist in those other services after having finished to serve the groups*. *The group activities shouldn’t be interrupted*.

(FG3 R8)

Two providers suggested that “there should be a permanent staff member for group care alone.” (FG2 R2) Others are able to exercise more control over staff allocation because they have management roles:

*[Group care] adds to our workload as others have said*, *but I am lucky because it is me who plans the work to be done*. *Therefore I allot enough time to it; and when it is necessary*, *I have a midwife who keeps on running the group care activities while I have to go to give a hand in a delivery case*. *We make sacrifices and allot duties to every person so that the work is done without a lot of problems*.

(FG 2 R5)

The second most common implementation problem is scheduling many group visits on 1 or 2 days each week even though a large number of pregnant women seek services. For example:

*You may find yourself handling three group care visits in the morning*, *You take little time to talk briefly to the members of group care 2*, *then group 3 and do the same for group 4*. *You don’t have time to deepen the matter; the results may not be good 100%*.

(FG 3 R4)

Peers in the FGD suggested that the health center should plan for just “one group in the morning … [and] another in the afternoon and you will manage to serve them all correctly and satisfactorily” (FG2 R5). In order to achieve this, providers encouraged one another to be proactive and advocate for a work schedule that allows for group ANC on most days of the week.

*I have registers where I plan my work throughout the entire year*. *I present my plan every month so that the management will know where I will be working from*. *Yes of course all may depend upon the health center; but when I have presented my plan of activities*, *they elaborate a timetable taking into account that plan of mine*. *For example*, *they know that from Tuesday to Friday I work with group care for antenatal consultation services*.

(FG1 R9)

Another provider was impressed by the statement above and immediately responded:

*I have learnt also to play a role in boldly speaking to the manager in favor of group care when elaborating the timetable*. *We shall inform them about how the group care activities are scheduled throughout the week so that they will provide room for the people trained to handle group care and do that very job without having much work in other services*.

(FG1 R4)

These FGDs supplied data while simultaneously providing opportunites for participants to learn from one another and adjust their plans and policies.

Finally, providers acknowledge that there are still barriers to women’s participation in group ANC/PNC: long distances from home to the health center, delays at the insurance payment office that delay the start of ANC/PNC visits, and poor attendance in general at the 6-week PNC visit. Solutions to these challenges suggested by providers during the FGDs include:

- Organize women who live close to the health center into groups that meet during the morning and women who live far from the health center into groups that meet in the afternoon (FG3 R1);
- Cooperate with the insurance payment office so they will allow women to pay after the visit rather than require payment before the group visit begins (FG3 R6, FG2 R1); and

Plan for group PNC on the same days women present for newborn immunizations (FG2 R10).

## Discussion

These results from group ANC and PNC providers in Rwanda are consistent with other published reports of provider reactions in other countries. A repeating theme across qualitative studies is that group ANC offers providers an opportunity to share more knowledge and support with pregnant women, and that this opportunity results in increased pride in their work. [10,13,20] Another common finding across these studies is that providers perceive that their workload increased when they implement group ANC, largely related to increased organizational tasks and spending significantly more time with women.[10,12] We found that our results shared a common theme with a study of group ANC in Malawi and Tanzania: group care allowed women to speak openly about their uses of traditional medicine to self-treat for symptoms that may require facility-based treatments.[21] Our results suggest that group ANC and PNC discussions persuade women to avoid traditional medicine use during pregnancy and for their newborns after birth.

In this study, results related to job satisfaction were mixed. Some focus group participants reported that delivering group ANC/PNC resulted in an increase in the quality of care they could offer, while others reported that when they are required to cover multiple services at once the group ANC/PNC participants may be disappointed. Some reported increased job satisfaction and pride in their work, while others reported being so overwhelmed by large numbers of women scheduled for group care on a single day of the week that they could not “serve them as well” as expected. The quantitative results are consistent with this mixed response. Job satisfaction related to ANC/PNC was similar across all groups described here: providers at both group care and individual care study sites and focus group participants. Even though job satisfaction among group care providers was not higher at follow-up, at the follow-up time point 86% of them preferred delivering group ANC/PNC to individual ANC/PNC. Our explanation is that while most providers appreciate the benefits of group ANC/PNC provision for themselves and for mothers, the circumstances in which they are expected to provide group care may result in frustration and increased work load.

We did not collect data from CHWs who participate as co-facilitators of group care. We plan to conduct FGDs among CHWs at the end of the study period. We look forward to comparing their responses to those of nurses and midwives. The focus group guide used for the FGDs reported in this article did not specifically address the perspective of the nurses and midwives regarding co-facilitation with CHWs; this topic did not arise spontaneously in any of the FGDs.

When we brought providers from different health centers together to share their experiences in FGDs, two natural results of the discussion were story sharing and peer support. The group process afforded rich qualitative data about provider experiences with this new program, and the peer-to-peer sharing that occurred during the FGD may have served to motivate participants who were struggling to implement group ANC/PNC effectively. While providers likely benefit from the supportive supervision of Master Trainers who visit their facilities regularly, we believe that meeting with a group of peers and participating in a facilitated discussion about accomplishments and challenges is more likely to help participants solve the most difficult, context-specific group ANC/PNC implementation issues. We assert that stakeholders who plan for the implementation of group care programs should include scheduled group discussions across facilities as a key activity. The exact intervals at which these group discussions should occur is not known, but resources should be allocated to convene these meetings as often as is feasible. Building a community of practice among new group care facilitators through regular in-person discussions is likely to result in increased knowledge and self-efficacy among providers, just as group ANC can increase these among pregnant women.

The circumstances of the facilities within which group ANC and PNC were implemented for this study (the Rwanda public health system at the health center level) resulted in a specific complaint from providers that has not been documented in other published articles about group ANC. In the staffing model at these facilities, nurses and midwives must be willing and able to cover several different health center services, such as ANC, maternity, immunizations, family planning, or even pharmacy. At the beginning of each work day, if the facility is short of staff for any number of reasons, the facility director must allocate the available staff to cover all facility services. In most cases, the facility director assigns the same provider to group ANC or PNC and maternity (labor and delivery). As maternity care is an emergency service, providers will be frequently called to leave a group visit and attend women in childbirth. This results in disruptions of scheduled group visits which may annoy women and stress providers.

These staffing issues are likely not unique to Rwanda’s health centers. Health worker shortages are the global rule rather than the exception.[22] Group ANC/PNC offers women and providers closeness, caring, and community, but in order to successfully scale this model each health center must have a complete cadre of providers who are allocated according to the requirements of group care. The health center director must not only support group care but have the management skills to effectively plan for a qualified provider to lead every group visit—even when staff are absent for training, vacation, or sick days. Additional staff time is required to plan for work schedules that can accommodate group visits, organize pregnant women into groups, and interact meaningfully with women during care.

### Limitations

We did not include all providers trained in GANC in the 3 FGDs. Some important provider perspectives may be absent from our data set, particularly those of providers who “opted out” of GANC/GPNC participation by avoiding engagement with the program and shifting responsibility of its provision to the FGD participants represented here.

## Conclusion

While our results may not reflect the experiences of all providers at Rwandan health centers, these qualitative results offer valuable insights into the experiences of the nurses and midwives most actively involved in group ANC/PNC in Rwanda during the first year of implementation. There is a continuing need to understand staff workload and requirements in Rwanda; this is an important area for future research. These and other outcomes-focused results are under careful consideration by stakeholders and policy-makers as they consider whether or not group ANC and PNC can be spread to a larger number of health centers in Rwanda. We hope our results will be of use to policy makers in other contexts, who should expect that many women and providers will find group ANC/PNC advantageous and that implementation challenges, while inevitable, can be addressed by creative and empowered health workers practicing this innovative model of care.

## Supporting information

S1 Doc Appendix 1: Baseline Provider Questionnaire; Appendix 2: Follow-up Group Care Provider Questionnaire; Appendix 3: Follow-up Control Provider Questionnaire. (PDF)

S2 Doc Appendix 4: Focus group guide. (PDF)
