# Supplementary material for: Nurses’ and midwives’ experiences of providing group antenatal and postnatal care at 18 health centers in Rwanda: A mixed methods study
Source: PLoS One. 2019 Jul 11;14(7):e0219471. doi: 10.1371/journal.pone.0219471 (PMC6622527; doi:10.1371/journal.pone.0219471)
Supplement: S1 Doc — (PDF) [file pone.0219471.s001.pdf]

Questionnaire Base du Prestataire/Baseline Provider Questionnaire

Code-fiche

____
(Record ID)

1. Code-prestataire

____
(Provider Code)

2. Indiquer la date du jour (cliquer le bouton "Today")

____
(Please indicate today's date (click the "Today" button))

Caractéristiques descriptives/Descriptive characteristics

3. Quel âge as-tu?

____
(How old are you?)

4. Quel est votre titre professionnel?
(What is your professional title?)

◯ Infirmière/Nurse
◯ Sage-femme/Midwife
◯ Autre/Other

4a. Autre:

____
(Other)

5. Votre établissement de santé offre-t-il aujourd'hui des services de soins prénatals et postnatals?
(Do you currently provide ANC or PNC in your health facility?)

◯ Soins prénatals uniquement/ANC only
◯ Soins postnatals uniquement/PNC only
◯ Soins prénatals et postnatals à la fois/Both ANC and PNC
◯ Ni soins prénatals ni soins postnatals/Neither ANC nor PNC

6. Quel est le plus haut niveau de scolarité ou de formation que tu as complété?
(What is your highest level of education or training?)

◯ École primaire/Primary School
◯ Formation professionnelle post-primaire/Post-primary vocational training
◯ École secondaire/Secondary school
◯ Formation professionnelle postsecondaire/Post-secondary vocational training
◯ Université/University

6a. Combien d'années d'études primaires as-tu complétées?

____
(How many years of primary school did you complete?)

6b. Combien d'années de formation professionnelle post-primaire as-tu complétées?

____
(How many years of post-primary vocational training did you complete?)

6c. Combien d'années d'études secondaires as-tu complétées?

____
(How many years of secondary school did you complete?)

6d. Combien d'années de formation professionnelle post-secondaire as-tu complétées?

____
(How many years of post-secondary vocational training did you complete?)

6e. Combien d'années d'études universitaires as-tu complétées?

____
(How many years of university did you complete?)

7. Depuis combien d'années occupes-tu un poste dont les tâches consistent à offrir des services de soins prénatals et/ou postnatals? (Veuillez saisir le nombre d'années.)

____
(How many years have you been working in a position in which you provided ANC and/or PNC? (Please enter the number of years.))

8. Depuis combien d'années travailles-tu dans l'établissement de santé pour lequel tu travailles actuellement? (Veuillez saisir le nombre d'années.)

____
(How many years have you worked in the health facility where you are currently employed? (Please enter the number of years.))

9. Dans quel service de l'établissement de santé préfères-tu travailler? (Veuillez choisir votre réponse.)
(In which area of the health facility do you most prefer to work? (Please choose your top answer.))

◯ Soins prénatals/ANC
◯ Consultation postnatale/PNC
◯ Maternité, Travail et accouchement/Maternity, Labor & Delivery
◯ Planification familiale/Family Planning
◯ Vaccinations/Immunizations
◯ Surveillance de la croissance et Réhabilitation nutritionnelle thérapeutique/Growth Monitoring and Therapeutic nutritional rehabilitation
◯ Intervention chirurgicale mineure/Minor surgery
◯ Services de soins palliatifs/Palliative care
◯ Consultation prénuptiale/Pre-marital consultation
◯ VCT, PTME et dépistage à l'initiative du prestataire (PIT)/VCT, PMTCT and PIT
◯ Consultation primaire curative/Primary curative consultation
◯ Soins et traitement pour les personnes vivant avec le VIH/SIDA--Care and treatment for person living with HIV/AIDS
◯ Hospitalisation/Hospitalization
◯ Services de stockage et de fourniture de médicaments/Drug storage & Supply services
◯ Position de leadership (Planification, S & E, Gestion du personnel ...)/Leadership position (Planning, M&E, Personnel management ...)
◯ Autre (veuillez préciser)/Other (please specify)

9a. Autre service de l'établissement de santé dans lequel tu préfères travailler:

____
(Other area in the health facility where you most prefer to work:)

---

10. Quel est ton deuxième choix quant au service de l'établissement de santé dans lequel tu préfères travailler?
(What is your second choice for an area of the health facility in which you prefer to work?)

◯ Soins prénatals/ANC
◯ Consultation postnatale/PNC
◯ Maternité, Travail et accouchement/Maternity, Labor & Delivery
◯ Planification familiale/Family Planning
◯ Vaccinations/Immunizations
◯ Surveillance de la croissance et Réhabilitation nutritionnelle thérapeutique/Growth Monitoring and Therapeutic nutritional rehabilitation
◯ Intervention chirurgicale mineure/Minor surgery
◯ Services de soins palliatifs/Palliative care
◯ Consultation prénuptiale/Pre-marital consultation
◯ VCT, PTME et dépistage à l'initiative du prestataire (PIT)/VCT, PMTCT and PIT
◯ Consultation primaire curative/Primary curative consultation
◯ Soins et traitement pour les personnes vivant avec le VIH/SIDA--Care and treatment for person living with HIV/AIDS
◯ Hospitalisation/Hospitalization
◯ Services de stockage et de fourniture de médicaments/Drug storage & Supply services
◯ Position de leadership (Planification, S & E, Gestion du personnel ...)/Leadership position (Planning, M&E, Personnel management ...)
◯ Autre (veuillez préciser)/Other (please specify)

10a. Autre service de l'établissement de santé dans lequel tu préfères travailler (deuxième choix):

____
(Other area in the health facility where you prefer to work (second choice):)

11. Quel est ton troisième choix quant au service de l'établissement de santé dans lequel tu préfères travailler?
(What is your third choice for an area of the health facility in which you prefer to work?)

◯ Soins prénatals/ANC
◯ Consultation postnatale/PNC
◯ Maternité, Travail et accouchement/Maternity, Labor & Delivery
◯ Planification familiale/Family Planning
◯ Vaccinations/Immunizations
◯ Surveillance de la croissance et Réhabilitation nutritionnelle thérapeutique/Growth Monitoring and Therapeutic nutritional rehabilitation
◯ Intervention chirurgicale mineure/Minor surgery
◯ Services de soins palliatifs/Palliative care
◯ Consultation prénuptiale/Pre-marital consultation
◯ VCT, PTME et dépistage à l'initiative du prestataire (PIT)/VCT, PMTCT and PIT
◯ Consultation primaire curative/Primary curative consultation
◯ Soins et traitement pour les personnes vivant avec le VIH/SIDA--Care and treatment for person living with HIV/AIDS
◯ Hospitalisation/Hospitalization
◯ Services de stockage et de fourniture de médicaments/Drug storage & Supply services
◯ Position de leadership (Planification, S & E, Gestion du personnel ...)/Leadership position (Planning, M&E, Personnel management ...)
◯ Autre (veuillez préciser)/Other (please specify)

11a. Autre secteur de l'établissement de santé dans lequel tu préfères travailler (troisième choix):

____
(Other area of the health facility in which you prefer to work (third choice):)

Satisfaction professionnelle/Job Satisfaction

En réfléchissant plus particulièrement aux services de soins prénatals et/ou postnatals que tu offres, peux-tu dire que tu es Fortement en accord, en Accord, ni en Accord ni en Désaccord, en Désaccord ou Fortement en désaccord avec chacune de ces affirmations?

12. Je suis en mesure d'offrir le niveau de qualité des soins que je suis censé offrir
(I am able to provide the level of quality of care that I am expected to provide)

◯ Fortement en accord/Strongly agree
◯ en Accord/Agree
◯ ni en Accord ni en Désaccord/Neither agree nor disagree
◯ en Désaccord/Disagree
◯ Fortement en désaccord/Strongly disagree

---

13. Pendant les consultations, j'ai suffisamment de temps à passer avec mes patients
(During visits I have enough time with my patients)

◯ Fortement en accord/Strongly agree
◯ en Accord/Agree
◯ ni en Accord ni en Désaccord/Neither agree nor disagree
◯ en Désaccord/Disagree
◯ Fortement en désaccord/Strongly disagree

14. Je suis en mesure de répondre aux questions dans un langage compris par les femmes
(I am able to answer questions in a way the women understand)

◯ Fortement en accord/Strongly agree
◯ en Accord/Agree
◯ ni en Accord ni en Désaccord/Neither agree nor disagree
◯ en Désaccord/Disagree
◯ Fortement en désaccord/Strongly disagree

15. Je me sens respecté par les femmes auxquelles j'offre les soins
(I feel respected by the women to whom I deliver care)

◯ Fortement en accord/Strongly agree
◯ en Accord/Agree
◯ ni en Accord ni en Désaccord/Neither agree nor disagree
◯ en Désaccord/Disagree
◯ Fortement en désaccord/Strongly disagree

16. Pendant les soins prénatals et / ou postnatals, je dispose de suffisamment de temps et suis en mesure de délivrer d'importants messages permettant aux femmes de se maintenir et de maintenir leurs bébés en bonne santé
(During antenatal and/or postnatal care I have the time and am able to teach important information about how women can keep themselves and their babies healthy)

◯ Fortement en accord/Strongly agree
◯ en Accord/Agree
◯ ni en Accord ni en Désaccord/Neither agree nor disagree
◯ en Désaccord/Disagree
◯ Fortement en désaccord/Strongly disagree

17. Je recommanderais à une amie de venir à ma clinique pour des soins prénatals ou postnatals
(I would recommend to a friend to go to my clinic for antenatal or postnatal care)

◯ Fortement en accord/Strongly agree
◯ en Accord/Agree
◯ ni en Accord ni en Désaccord/Neither agree nor disagree
◯ en Désaccord/Disagree
◯ Fortement en désaccord/Strongly disagree

18. Parfois, il m'arrive de craindre que les patients retournent à la maison avec des questions sans réponses ou confuses au sujet de certaines choses
(Sometimes I worry that patients leave their visits with unanswered questions or confused about some things)

◯ Fortement en accord/Strongly agree
◯ en Accord/Agree
◯ ni en Accord ni en Désaccord/Neither agree nor disagree
◯ en Désaccord/Disagree
◯ Fortement en désaccord/Strongly disagree

19. Je suis toujours en mesure de me montrer respectueuse envers mes patients pendant les consultations
(I am always able to be respectful towards my patients during visits)

◯ Fortement en accord/Strongly agree
◯ en Accord/Agree
◯ ni en Accord ni en Désaccord/Neither agree nor disagree
◯ en Désaccord/Disagree
◯ Fortement en désaccord/Strongly disagree

20. En réfléchissant plus particulièrement aux jours où tu offres des services de soins prénatals ou postnatals, sur une échelle de 1 à 5, 1 étant extrêmement insatisfait et 5 extrêmement satisfait, comment évalueriez-vous votre degré de satisfaction professionnelle actuelle?
(how would you rate your current job satisfaction?)

◯ 1 Extrêmement insatisfait/Extremely dissatisfied
◯ 2 Insatisfait/Dissatisfied
◯ 3 Neutre/Neutral
◯ 4 Satisfait/Satisfied
◯ 5 Extrêmement satisfait/Extremely satisfied

Motivation du prestataire/Provider motivation

En réfléchissant plus particulièrement à votre travail dans son ensemble, y compris toutes vos tâches, peux-tu dire que tu es Fortement en accord, en Accord, ni en Accord ni en Désaccord, en Désaccord ou Fortement en désaccord avec chacune de ces affirmations?
21. Je me sens motivé à travailler aussi dur que je peux
(I feel motivated to work as hard as I can)

◯ Fortement en accord/Strongly agree
◯ en Accord/Agree
◯ ni en Accord ni en Désaccord/Neither agree nor disagree
◯ en Désaccord/Disagree
◯ Fortement en désaccord/Strongly disagree

22. La seule raison pour laquelle je fais ce travail est d'être payée
(The only reason I do this job is to get paid)

◯ Fortement en accord/Strongly agree
◯ en Accord/Agree
◯ ni en Accord ni en Désaccord/Neither agree nor disagree
◯ en Désaccord/Disagree
◯ Fortement en désaccord/Strongly disagree

23. Je me sens vidée de ma force émotionnelle à la fin de chaque jour
(I feel emotionally drained at the end of every day)

◯ Fortement en accord/Strongly agree
◯ en Accord/Agree
◯ ni en Accord ni en Désaccord/Neither agree nor disagree
◯ en Désaccord/Disagree
◯ Fortement en désaccord/Strongly disagree

24. Parfois, quand je me lève le matin, je redoute d'avoir à faire face à un autre jour au travail
(Sometimes when I get up in the morning, I dread having to face another day at work)

◯ Fortement en accord/Strongly agree
◯ en Accord/Agree
◯ ni en Accord ni en Désaccord/Neither agree nor disagree
◯ en Désaccord/Disagree
◯ Fortement en désaccord/Strongly disagree

25. J'accomplis des tâches qui doivent être faites sans que l'on me le demande ou dit
(I do things that need doing without being asked or told)

◯ Fortement en accord/Strongly agree
◯ en Accord/Agree
◯ ni en Accord ni en Désaccord/Neither agree nor disagree
◯ en Désaccord/Disagree
◯ Fortement en désaccord/Strongly disagree

26. Ce n'est pas grave si j'arrive parfois tard au travail
(It is not a problem if I sometimes come late to work)

◯ Fortement en accord/Strongly agree
◯ en Accord/Agree
◯ ni en Accord ni en Désaccord/Neither agree nor disagree
◯ en Désaccord/Disagree
◯ Fortement en désaccord/Strongly disagree

27. Je me sens fort peu engagé envers l'établissement de santé pour lequel je travaille
(I feel very little commitment to the health facility where I work)

◯ Fortement en accord/Strongly agree
◯ en Accord/Agree
◯ ni en Accord ni en Désaccord/Neither agree nor disagree
◯ en Désaccord/Disagree
◯ Fortement en désaccord/Strongly disagree

Opinions du prestataire/Provider opinions

En réfléchissant à ton expérience en matière d'offre de soins aux femmes enceintes et aux nouvelles mères, peux-tu dire que tu es Fortement en accord, en Accord, ni en Accord ni en Désaccord, en Désaccord ou Fortement en désaccord avec chacune de ces affirmations?

28. Il faut apprendre aux femmes enceintes et aux nouvelles mères la façon de prendre soin d'elles-mêmes car elles ont du mal à comprendre les comportements sains
(Pregnant and new mothers must be told how to care for themselves because they do not understand healthy behaviors)

◯ Fortement en accord/Strongly agree
◯ en Accord/Agree
◯ ni en Accord ni en Désaccord/Neither agree nor disagree
◯ en Désaccord/Disagree
◯ Fortement en désaccord/Strongly disagree

29. Les femmes enceintes ont de bonnes idées sur la façon de se maintenir en bonne santé
(Pregnant mothers have good ideas about how to keep themselves healthy)

◯ Fortement en accord/Strongly agree
◯ en Accord/Agree
◯ ni en Accord ni en Désaccord/Neither agree nor disagree
◯ en Désaccord/Disagree
◯ Fortement en désaccord/Strongly disagree

30. J'aime demander aux patients de partager leurs opinions, leurs histoires ou leurs idées
(I like to ask patients about their opinions, stories, or ideas)

◯ Fortement en accord/Strongly agree
◯ en Accord/Agree
◯ ni en Accord ni en Désaccord/Neither agree nor disagree
◯ en Désaccord/Disagree
◯ Fortement en désaccord/Strongly disagree

En réfléchissant à tes propres préférences personnelles, peux-tu dire que tu es Fortement en accord, en Accord, ni en Accord ni en Désaccord, en Désaccord ou Fortement en désaccord avec chacune de ces affirmations?
31. J'ai le désir d'essayer de nouvelles façons de faire des choses plutôt que de toujours faire les choses de la même manière
(I would like to try new ways of doing things rather than always doing things the same way)

◯ Fortement en accord/Strongly agree
◯ en Accord/Agree
◯ ni en Accord ni en Désaccord/Neither agree nor disagree
◯ en Désaccord/Disagree
◯ Fortement en désaccord/Strongly disagree

32. Si j'ai plus de temps, je préfère le consacrer à une activité calme que je fais toute seule
(If I have extra time, I prefer to spend that time in a quiet activity by myself)

◯ Fortement en accord/Strongly agree
◯ en Accord/Agree
◯ ni en Accord ni en Désaccord/Neither agree nor disagree
◯ en Désaccord/Disagree
◯ Fortement en désaccord/Strongly disagree

33. Si j'ai plus de temps, j'aime le consacrer à la rencontre avec de grands groupes d'amis ou de famille
(If I have extra time, I like to spend that time with large groups of friends or family)

◯ Fortement en accord/Strongly agree
◯ en Accord/Agree
◯ ni en Accord ni en Désaccord/Neither agree nor disagree
◯ en Désaccord/Disagree
◯ Fortement en désaccord/Strongly disagree

34. Chaque personne que je rencontre, je la considère comme mon égale, même si nous sommes très différents
(Every person I meet is equal to me even if we are very different)

◯ Fortement en accord/Strongly agree
◯ en Accord/Agree
◯ ni en Accord ni en Désaccord/Neither agree nor disagree
◯ en Désaccord/Disagree
◯ Fortement en désaccord/Strongly disagree

Échelle du stress ressenti/Perceived Stress Scale

Ces questions permettent d'identifier les émotions et les pensées que tu as affichées au cours du dernier mois.

35. Au cours du dernier mois, à quelle fréquence t'es-tu senti bouleversé en raison d'un événement imprévu?
(In the last month, how often have you been upset because of something that happened unexpectedly?)

◯ Jamais/Never
◯ Rarement/Almost never
◯ Parfois/Sometimes
◯ Très souvent/Fairly often
◯ Toujours/Very often

36. Au cours du dernier mois, à quelle fréquence as-tu senti que tu n'étais pas en mesure de maîtriser les éléments importants de ta vie?
(In the last month, how often have you felt that you were unable to control the important things in your life?)

◯ Jamais/Never
◯ Rarement/Almost never
◯ Parfois/Sometimes
◯ Très souvent/Fairly often
◯ Toujours/Very often

37. Au cours du dernier mois, à quelle fréquence t'es-tu senti nerveux et "stressé"?
(In the last month, how often have you felt nervous and "stressed"?)

◯ Jamais/Never
◯ Rarement/Almost never
◯ Parfois/Sometimes
◯ Très souvent/Fairly often
◯ Toujours/Very often

38. Au cours du dernier mois, à quelle fréquence t'es-tu senti confiant quant à ta capacité de gérer tes problèmes personnels?
(In the last month, how often have you felt confident about your ability to handle your personal problems?)

◯ Jamais/Never
◯ Rarement/Almost never
◯ Parfois/Sometimes
◯ Très souvent/Fairly often
◯ Toujours/Very often

39. Au cours du dernier mois, à quelle fréquence t'es-tu senti que tu ne pouvais pas prendre en charge toutes les choses que tu devais faire?
(In the last month, how often have you found that you could not cope with all the things that you had to do?)

◯ Jamais/Never
◯ Rarement/Almost never
◯ Parfois/Sometimes
◯ Très souvent/Fairly often
◯ Toujours/Very often

40. Au cours du dernier mois, à quelle fréquence t'es-tu senti que les choses allaient comme tu le voulais?
(In the last month, how often have you felt that things were going your way?)

◯ Jamais/Never
◯ Rarement/Almost never
◯ Parfois/Sometimes
◯ Très souvent/Fairly often
◯ Toujours/Very often

41. Au cours du dernier mois, à quelle fréquence as-tu pu être en mesure de contrôler les irritations dans ta vie?
(In the last month, how often have you been able to control irritations in your life?)

◯ Jamais/Never
◯ Rarement/Almost never
◯ Parfois/Sometimes
◯ Très souvent/Fairly often
◯ Toujours/Very often

42. Au cours du dernier mois, à quelle fréquence as-tu senti que tu prenais le dessus sur tout?
(In the last month, how often have you felt that you were on top of things?)

◯ Jamais/Never
◯ Rarement/Almost never
◯ Parfois/Sometimes
◯ Très souvent/Fairly often
◯ Toujours/Very often

43. Au cours du dernier mois, à quelle fréquence t'es-tu mis en colère en raison des choses qui échappaient à ton contrôle?
(In the last month, how often have you been angered because of things that were outside of your control?)

◯ Jamais/Never
◯ Rarement/Almost never
◯ Parfois/Sometimes
◯ Très souvent/Fairly often
◯ Toujours/Very often

44. Au cours du dernier mois, combien de fois as-tu senti que les difficultés prenaient tellement de place que tu ne pourrais pas toutes les surmonter?
(In the last month, how often have you felt difficulties were piling up so high that you could not overcome them?)

◯ Jamais/Never
◯ Rarement/Almost never
◯ Parfois/Sometimes
◯ Très souvent/Fairly often
◯ Toujours/Very often

Questionnaire Périodique du Prestataire: Modèle de soins en groupe/Follow-up Provider Questionnaire: Group Care

Code-fiche

____
(Record ID)

1. Code-prestataire

____
(Provider Code)

2. Indiquer la date du jour (cliquer le bouton "Today")

____
(Please indicate today's date (click the "Today" button))

Expérience du prestataire/Provider experience

3. As-tu servi de co-facilitateur dans le modèle du groupe de soins prénatals/postnatals ? Ce modèle de soins de groupe est également connu sous le nom d' "Ibarukeneza Mubyeyi."

(Have you been a co-facilitator in the group ANC/PNC model?)

◯ Oui/Yes
◯ Non/No

3a. Explique pourquoi votre centre de santé n'a pas offert des soins de groupe après avoir été formé en intervention de soins en groupe.

(Please explain why you did not provide group care at your health center after you were trained in the group care intervention.)

4. Selon ton estimation, combien de visites de groupe au total dans lesquelles tu as participé en tant que prestataire/cofacilitateur

____
(In your best estimation, how many total group visits have you participated in as a provider/co-facilitator?)

Satisfaction professionnelle/Job satisfaction

En réfléchissant plus particulièrement au groupe de soins prénatals ou postnatals dans lequel tu sers de prestataire dans le cadre du modèle de soins en groupe ou du modèle Ibarukeneza Mubyeyi, peux-tu dire que tu es Fortement en accord, en Accord, ni en Accord ni en Désaccord, en Désaccord ou Fortement en désaccord avec chacune de ces affirmations?
5. Je suis en mesure d'offrir le niveau de qualité des soins que je suis censé offrir

(Thinking specifically about the group ANC or PNC you provide in the group care model, or Ibarukeneza Mubyeyi model, do you Strongly agree, Agree, Neither agree nor disagree, Disagree, or Strongly disagree with each of these statements? 5. I am able to provide the level of quality of care that I am expected to provide)

◯ Fortement en accord/Strongly agree
◯ en Accord/Agree
◯ ni en Accord ni en Désaccord/Neither agree nor disagree
◯ en Désaccord/Disagree
◯ Fortement en désaccord/Strongly disagree

En réfléchissant plus particulièrement aux services de soins prénatals et/ou postnatals que tu offres, peux-tu dire que tu es Fortement en accord, en Accord, ni en Accord ni en Désaccord, en Désaccord ou Fortement en désaccord avec chacune de ces affirmations?
5. Je suis en mesure d'offrir le niveau de qualité des soins que je suis censé offrir

(Thinking specifically about the ANC and/or PNC you provide, do you Strongly agree, Agree, Neither agree nor disagree, Disagree, or Strongly disagree with each of these statements? 5. I am able to provide the level of quality of care that I am expected to provide)

◯ Fortement en accord/Strongly agree
◯ en Accord/Agree
◯ ni en Accord ni en Désaccord/Neither agree nor disagree
◯ en Désaccord/Disagree
◯ Fortement en désaccord/Strongly disagree

---

6. Pendant les consultations, j'ai suffisamment de temps à passer avec mes patients

(During visits I have enough time with my patients)

◯ Fortement en accord/Strongly agree
◯ en Accord/Agree
◯ ni en Accord ni en Désaccord/Neither agree nor disagree
◯ en Désaccord/Disagree
◯ Fortement en désaccord/Strongly disagree

---

7. Je suis en mesure de répondre aux questions dans un langage compris par les femmes
(I am able to answer questions in a way the women understand)

◯ Fortement en accord/Strongly agree
◯ en Accord/Agree
◯ ni en Accord ni en Désaccord/Neither agree nor disagree
◯ en Désaccord/Disagree
◯ Fortement en désaccord/Strongly disagree

---

8. Je me sens respecté par les femmes auxquelles j'offre les soins

(I feel respected by the women to whom I deliver care)

◯ Fortement en accord/Strongly agree
◯ en Accord/Agree
◯ ni en Accord ni en Désaccord/Neither agree nor disagree
◯ en Désaccord/Disagree
◯ Fortement en désaccord/Strongly disagree

---

9. Pendant les soins prénatals et / ou postnatals, je dispose de suffisamment de temps et suis en mesure de délivrer d'importants messages permettant aux femmes de se maintenir et de maintenir leurs bébés en bonne santé

(During antenatal and/or postnatal care I have the time and am able to teach important information about how women can keep themselves and their babies healthy)

◯ Fortement en accord/Strongly agree
◯ en Accord/Agree
◯ ni en Accord ni en Désaccord/Neither agree nor disagree
◯ en Désaccord/Disagree
◯ Fortement en désaccord/Strongly disagree

---

10. Je recommanderais à une amie de venir à ma clinique pour des soins prénatals ou postnatals

(I would recommend to a friend to go to my clinic for antenatal or postnatal care)

◯ Fortement en accord/Strongly agree
◯ en Accord/Agree
◯ ni en Accord ni en Désaccord/Neither agree nor disagree
◯ en Désaccord/Disagree
◯ Fortement en désaccord/Strongly disagree

---

11. Parfois, il m'arrive de craindre que les patients retournent à la maison avec des questions sans réponses ou confuses au sujet de certaines choses

(Sometimes I worry that patients leave their visits with unanswered questions or confused about some things)

◯ Fortement en accord/Strongly agree
◯ en Accord/Agree
◯ ni en Accord ni en Désaccord/Neither agree nor disagree
◯ en Désaccord/Disagree
◯ Fortement en désaccord/Strongly disagree

---

12. Je suis toujours en mesure de me montrer respectueuse envers mes patients pendant les consultations
(I am always able to be respectful towards my patients during visits)

◯ Fortement en accord/Strongly agree
◯ en Accord/Agree
◯ ni en Accord ni en Désaccord/Neither agree nor disagree
◯ en Désaccord/Disagree
◯ Fortement en désaccord/Strongly disagree

---

13. En réfléchissant plus particulièrement aux jours où tu offres des services de soins prénatals ou postnatals, sur une échelle de 1 à 5, 1 étant extrêmement insatisfait et 5 extrêmement satisfait, comment évalueriez-vous votre degré de satisfaction professionnelle actuelle?

(Thinking specifically about the days you provide ANC or PNC care, on a scale of 1-5, with 1 being extremely dissatisfied and 5 being extremely satisfied, how would you rate your current job satisfaction?)

◯ 1 Extrêmement insatisfait/Extremely dissatisfied
◯ 2 Insatisfait/Dissatisfied
◯ 3 Neutre/Neutral
◯ 4 Satisfait/Satisfied
◯ 5 Extrêmement satisfait/Extremely satisfied

14. Comment prèfèrez-vous fournir des soins prènatals et/ou postnatals?
(How do you prefer to provide antenatal and/or postnatal care?)

◯ Visites de soins individuels, comme cela a ete fait avant l'introduction du modèle de soins en groupe/Individual visits only, as was done before the group care model was introduced
◯ Modèle de soins en groupe (Ibarukeneza Mubyeyi)/Group care model (also known as Ibarukeneza Mubyeyi)
◯ Le deux ègalement/Both equally
◯ Indecis/Undecided

Motivation du prestataire/Provider motivation

En réfléchissant plus particulièrement à votre travail dans son ensemble, y compris toutes vos tâches, peux-tu dire que tu es Fortement en accord, en Accord, ni en Accord ni en Désaccord, en Désaccord ou Fortement en désaccord avec chacune de ces affirmations?
15. Je me sens motivé à travailler aussi dur que je peux

(I feel motivated to work as hard as I can)

◯ Fortement en accord/Strongly agree
◯ en Accord/Agree
◯ ni en Accord ni en Désaccord/Neither agree nor disagree
◯ en Désaccord/Disagree
◯ Fortement en désaccord/Strongly disagree

16. La seule raison pour laquelle je fais ce travail est d'être payée

(The only reason I do this job is to get paid)

◯ Fortement en accord/Strongly agree
◯ en Accord/Agree
◯ ni en Accord ni en Désaccord/Neither agree nor disagree
◯ en Désaccord/Disagree
◯ Fortement en désaccord/Strongly disagree

17. Je me sens vidée de ma force émotionnelle à la fin de chaque jour

(I feel emotionally drained at the end of every day)

◯ Fortement en accord/Strongly agree
◯ en Accord/Agree
◯ ni en Accord ni en Désaccord/Neither agree nor disagree
◯ en Désaccord/Disagree
◯ Fortement en désaccord/Strongly disagree

18. Parfois, quand je me lève le matin, je redoute d'avoir à faire face à un autre jour au travail

(Sometimes when I get up in the morning, I dread having to face another day at work)

◯ Fortement en accord/Strongly agree
◯ en Accord/Agree
◯ ni en Accord ni en Désaccord/Neither agree nor disagree
◯ en Désaccord/Disagree
◯ Fortement en désaccord/Strongly disagree

19. J'accomplis des tâches qui doivent être faites sans que l'on me le demande ou dit

(I do things that need doing without being asked or told)

◯ Fortement en accord/Strongly agree
◯ en Accord/Agree
◯ ni en Accord ni en Désaccord/Neither agree nor disagree
◯ en Désaccord/Disagree
◯ Fortement en désaccord/Strongly disagree

20. Ce n'est pas grave si j'arrive parfois tard au travail

(It is not a problem if I sometimes come late to work)

◯ Fortement en accord/Strongly agree
◯ en Accord/Agree
◯ ni en Accord ni en Désaccord/Neither agree nor disagree
◯ en Désaccord/Disagree
◯ Fortement en désaccord/Strongly disagree

21. Je me sens fort peu engagé envers l'établissement de santé pour lequel je travaille

(I feel very little commitment to the health facility where I work)

◯ Fortement en accord/Strongly agree
◯ en Accord/Agree
◯ ni en Accord ni en Désaccord/Neither agree nor disagree
◯ en Désaccord/Disagree
◯ Fortement en désaccord/Strongly disagree

Opinions du prestataire/Provider opinions

En réfléchissant à ton expérience en matière d'offre de soins aux femmes enceintes et aux nouvelles mères, peux-tu dire que tu es Fortement en accord, en Accord, ni en Accord ni en Désaccord, en Désaccord ou Fortement en désaccord avec chacune de ces affirmations?

22. Il faut apprendre aux femmes enceintes et aux nouvelles mères la façon de prendre soin d'elles-mêmes car elles ont du mal à comprendre les comportements sains

(Pregnant and new mothers must be told how to care for themselves because they do not understand healthy behaviors)

◯ Fortement en accord/Strongly agree
◯ en Accord/Agree
◯ ni en Accord ni en Désaccord/Neither agree nor disagree
◯ en Désaccord/Disagree
◯ Fortement en désaccord/Strongly disagree

23. Les femmes enceintes ont de bonnes idées sur la façon de se maintenir en bonne santé

(Pregnant mothers have good ideas about how to keep themselves healthy)

◯ Fortement en accord/Strongly agree
◯ en Accord/Agree
◯ ni en Accord ni en Désaccord/Neither agree nor disagree
◯ en Désaccord/Disagree
◯ Fortement en désaccord/Strongly disagree

24. J'aime poser aux patients de partager leurs opinions, leurs histoires ou leurs idées

(I like to ask patients about their opinions, stories, or ideas)

◯ Fortement en accord/Strongly agree
◯ en Accord/Agree
◯ ni en Accord ni en Désaccord/Neither agree nor disagree
◯ en Désaccord/Disagree
◯ Fortement en désaccord/Strongly disagree

En réfléchissant à tes propres préférences personnelles, peux-tu dire que tu es Fortement en accord, en Accord, ni en Accord ni en Désaccord, en Désaccord ou Fortement en désaccord avec chacune de ces affirmations?

25. J'ai le désir d'essayer de nouvelles façons de faire des choses plutôt que de toujours faire les choses de la même manière

(I would like to try new ways of doing things rather than always doing things the same way)

◯ Fortement en accord/Strongly agree
◯ en Accord/Agree
◯ ni en Accord ni en Désaccord/Neither agree nor disagree
◯ en Désaccord/Disagree
◯ Fortement en désaccord/Strongly disagree

26. Chaque personne que je rencontre, je la considère comme mon égale, même si nous sommes très différents

(Every person I meet is equal to me even if we are very different)

◯ Fortement en accord/Strongly agree
◯ en Accord/Agree
◯ ni en Accord ni en Désaccord/Neither agree nor disagree
◯ en Désaccord/Disagree
◯ Fortement en désaccord/Strongly disagree

27. Dans quel service de l'établissement de santé préfères-tu travailler? (Veuillez choisir votre réponse.)

(What is the area in the health facility where you most prefer to work? (Please choose your top answer.))

◯ Soins prénatals/ANC
◯ Consultation postnatale/PNC
◯ Maternité, Travail et accouchement/Maternity, Labor & Delivery
◯ Planification familiale/Family Planning
◯ Vaccinations/Immunizations
◯ Surveillance de la croissance et Réhabilitation nutritionnelle thérapeutique/Growth Monitoring and Therapeutic nutritional rehabilitation
◯ Intervention chirurgicale mineure/Minor surgery
◯ Services de soins palliatifs/Palliative care
◯ Consultation prénuptiale/Pre-marital consultation
◯ VCT, PTME et dépistage à l'initiative du prestataire (PIT)/VCT, PMTCT and PIT
◯ Consultation primaire curative/Primary curative consultation
◯ Soins et traitement pour les personnes vivant avec le VIH/SIDA--Care and treatment for person living with HIV/AIDS
◯ Hospitalisation/Hospitalization
◯ Services de stockage et de fourniture de médicaments/Drug storage & Supply services
◯ Position de leadership (Planification, S & E, Gestion du personnel ...)/Leadership position (Planning, M&E, Personnel management ...)
◯ Autre (veuillez préciser)/Other (please specify)

27a. Autre service de l'établissement de santé dans lequel tu préfères travailler:

____

(Other area in the health facility where you most prefer to work (top choice):)

28. Quel est ton deuxième choix quant au service de l'établissement de santé dans lequel tu préfères travailler?

(What is your second choice for an area of the health facility in which you prefer to work?)

◯ Soins prénatals/ANC
◯ Consultation postnatale/PNC
◯ Maternité, Travail et accouchement/Maternity, Labor & Delivery
◯ Planification familiale/Family Planning
◯ Vaccinations/Immunizations
◯ Surveillance de la croissance et Réhabilitation nutritionnelle thérapeutique/Growth Monitoring and Therapeutic nutritional rehabilitation
◯ Intervention chirurgicale mineure/Minor surgery
◯ Services de soins palliatifs/Palliative care
◯ Consultation prénuptiale/Pre-marital consultation
◯ VCT, PTME et dépistage à l'initiative du prestataire (PIT)/VCT, PMTCT and PIT
◯ Consultation primaire curative/Primary curative consultation
◯ Soins et traitement pour les personnes vivant avec le VIH/SIDA--Care and treatment for person living with HIV/AIDS
◯ Hospitalisation/Hospitalization
◯ Services de stockage et de fourniture de médicaments/Drug storage & Supply services
◯ Position de leadership (Planification, S & E, Gestion du personnel ...)/Leadership position (Planning, M&E, Personnel management ...)
◯ Autre (veuillez préciser)/Other (please specify)

28a. Autre service de l'établissement de santé dans lequel tu préfères travailler (deuxième choix):

____
(Other area in the health facility where you prefer to work (second choice):)

29. Quel est ton troisième choix quant au service de l'établissement de santé dans lequel tu préfères travailler?

(What is your third choice for an area of the health facility in which you prefer to work?)

◯ Soins prénatals/ANC
◯ Consultation postnatale/PNC
◯ Maternité, Travail et accouchement/Maternity, Labor & Delivery
◯ Planification familiale/Family Planning
◯ Vaccinations/Immunizations
◯ Surveillance de la croissance et Réhabilitation nutritionnelle thérapeutique/Growth Monitoring and Therapeutic nutritional rehabilitation
◯ Intervention chirurgicale mineure/Minor surgery
◯ Services de soins palliatifs/Palliative care
◯ Consultation prénuptiale/Pre-marital consultation
◯ VCT, PTME et dépistage à l'initiative du prestataire (PIT)/VCT, PMTCT and PIT
◯ Consultation primaire curative/Primary curative consultation
◯ Soins et traitement pour les personnes vivant avec le VIH/SIDA--Care and treatment for person living with HIV/AIDS
◯ Hospitalisation/Hospitalization
◯ Services de stockage et de fourniture de médicaments/Drug storage & Supply services
◯ Position de leadership (Planification, S & E, Gestion du personnel ...)/Leadership position (Planning, M&E, Personnel management ...)
◯ Autre (veuillez préciser)/Other (please specify)

29a. Autre service de l'établissement de santé dans lequel tu préfères travailler (troisième choix):

____
(Other area in the health facility in which you most prefer to work (third choice):)

Échelle du stress ressenti/Perceived Stress Scale

Ces questions permettent d'identifier les émotions et les pensées que tu as affichées au cours du dernier mois.

30. Au cours du dernier mois, à quelle fréquence t'es-tu senti bouleversé en raison d'un événement imprévu?

(In the last month, how often have you been upset because of something that happened unexpectedly?)

◯ Jamais/Never
◯ Rarement/Almost never
◯ Parfois/Sometimes
◯ Très souvent/Fairly often
◯ Toujours/Very often

31. Au cours du dernier mois, à quelle fréquence as-tu senti que tu n'étais pas en mesure de maîtriser les éléments importants de ta vie ?

(In the last month, how often have you felt that you were unable to control the important things in your life?)

◯ Jamais/Never
◯ Rarement/Almost never
◯ Parfois/Sometimes
◯ Très souvent/Fairly often
◯ Toujours/Very often

32. Au cours du dernier mois, à quelle fréquence t'es-tu senti nerveux et "stressé"?

(In the last month, how often have you felt nervous and "stressed"?)

◯ Jamais/Never
◯ Rarement/Almost never
◯ Parfois/Sometimes
◯ Très souvent/Fairly often
◯ Toujours/Very often

33. Au cours du dernier mois, à quelle fréquence t'es-tu senti confiant quant à ta capacité de gérer tes problèmes personnels?
(In the last month, how often have you felt confident about your ability to handle your personal problems?)

◯ Jamais/Never
◯ Rarement/Almost never
◯ Parfois/Sometimes
◯ Très souvent/Fairly often
◯ Toujours/Very often

34. Au cours du dernier mois, à quelle fréquence t'es-tu senti que tu ne pouvais pas prendre en charge toutes les choses que tu devais faire?
(In the last month, how often have you found that you could not cope with all the things that you had to do?)

◯ Jamais/Never
◯ Rarement/Almost never
◯ Parfois/Sometimes
◯ Très souvent/Fairly often
◯ Toujours/Very often

35. Au cours du dernier mois, à quelle fréquence t'es-tu senti que les choses allaient comme tu le voulais?

(In the last month, how often have you felt that things were going your way?)

◯ Jamais/Never
◯ Rarement/Almost never
◯ Parfois/Sometimes
◯ Très souvent/Fairly often
◯ Toujours/Very often

36. Au cours du dernier mois, à quelle fréquence as-tu pu être en mesure de contrôler les irritations dans ta vie?

(In the last month, how often have you been able to control irritations in your life?)

◯ Jamais/Never
◯ Rarement/Almost never
◯ Parfois/Sometimes
◯ Très souvent/Fairly often
◯ Toujours/Very often

37. Au cours du dernier mois, à quelle fréquence as-tu senti que tu prenais le dessus sur tout ?

(In the last month, how often have you felt that you were on top of things?)

◯ Jamais/Never
◯ Rarement/Almost never
◯ Parfois/Sometimes
◯ Très souvent/Fairly often
◯ Toujours/Very often

38. Au cours du dernier mois, à quelle fréquence t'es-tu mis en colère en raison des choses qui échappaient à ton contrôle?

(In the last month, how often have you been angered because of things that were outside of your control?)

◯ Jamais/Never
◯ Rarement/Almost never
◯ Parfois/Sometimes
◯ Très souvent/Fairly often
◯ Toujours/Very often

39. Au cours du dernier mois, combien de fois as-tu senti que les difficultés prenaient tellement de place que tu ne pourrais pas toutes les surmonter ?

(In the last month, how often have you felt difficulties were piling up so high that you could not overcome them?)

◯ Jamais/Never
◯ Rarement/Almost never
◯ Parfois/Sometimes
◯ Très souvent/Fairly often
◯ Toujours/Very often

Questionnaire Périodique du Prestataire: Visites de soins individuels/Follow-up Provider Questionnaire: Control

Code-fiche

____
(Record ID)

1. Code-prestataire

____
(Provider Code)

2. Indiquer la date du jour (cliquer le bouton "Today")

____
(Indicate today's date (press the "Today" button))

Satisfaction professionnelle/Job satisfaction

En réfléchissant plus particulièrement aux services de soins prénatals et/ou postnatals que tu offres, peux-tu dire que tu es Fortement en accord, en Accord, ni en Accord ni en Désaccord, en Désaccord ou Fortement en désaccord avec chacune de ces affirmations?

3. Je suis en mesure d'offrir le niveau de qualité des soins que je suis censé offrir
(I am able to provide the level of quality of care that I am expected to provide)

◯ Fortement en accord/Strongly agree
◯ en Accord/Agree
◯ ni en Accord ni en Désaccord/Neither agree nor disagree
◯ en Désaccord/Disagree
◯ Fortement en désaccord/Strongly disagree

4. Pendant les consultations, j'ai suffisamment de temps à passer avec mes patients
(During visits I have enough time with my patients)

◯ Fortement en accord/Strongly agree
◯ en Accord/Agree
◯ ni en Accord ni en Désaccord/Neither agree nor disagree
◯ en Désaccord/Disagree
◯ Fortement en désaccord/Strongly disagree

5. Je suis en mesure de répondre aux questions dans un langage compris par les femmes
(I am able to answer questions in a way the women understand)

◯ Fortement en accord/Strongly agree
◯ en Accord/Agree
◯ ni en Accord ni en Désaccord/Neither agree nor disagree
◯ en Désaccord/Disagree
◯ Fortement en désaccord/Strongly disagree

6. Je me sens respecté par les femmes auxquelles j'offre les soins
(I feel respected by the women to whom I deliver care)

◯ Fortement en accord/Strongly agree
◯ en Accord/Agree
◯ ni en Accord ni en Désaccord/Neither agree nor disagree
◯ en Désaccord/Disagree
◯ Fortement en désaccord/Strongly disagree

---

7. Pendant les soins prénatals et / ou postnatals, je dispose de suffisamment de temps et suis en mesure de délivrer d'importants messages permettant aux femmes de se maintenir et de maintenir leurs bébés en bonne santé
(During antenatal and/or postnatal care I have the time and am able to teach important information about how women can keep themselves and their babies healthy)

◯ Fortement en accord/Strongly agree
◯ en Accord/Agree
◯ ni en Accord ni en Désaccord/Neither agree nor disagree
◯ en Désaccord/Disagree
◯ Fortement en désaccord/Strongly disagree

---

8. Je recommanderais à une amie de venir à ma clinique pour des soins prénatals ou postnatals
(I would recommend to a friend to go to my clinic for antenatal or postnatal care)

◯ Fortement en accord/Strongly agree
◯ en Accord/Agree
◯ ni en Accord ni en Désaccord/Neither agree nor disagree
◯ en Désaccord/Disagree
◯ Fortement en désaccord/Strongly disagree

---

9. Parfois, il m'arrive de craindre que les patients retournent à la maison avec des questions sans réponses ou confuses au sujet de certaines choses
(Sometimes I worry that patients leave their visits with unanswered questions or confused about some things)

◯ Fortement en accord/Strongly agree
◯ en Accord/Agree
◯ ni en Accord ni en Désaccord/Neither agree nor disagree
◯ en Désaccord/Disagree
◯ Fortement en désaccord/Strongly disagree

---

10. Je suis toujours en mesure de me montrer respectueuse envers mes patients pendant les consultations
(10. I am always able to be respectful towards my patients during visits)

◯ Fortement en accord/Strongly agree
◯ en Accord/Agree
◯ ni en Accord ni en Désaccord/Neither agree nor disagree
◯ en Désaccord/Disagree
◯ Fortement en désaccord/Strongly disagree

---

11. En réfléchissant plus particulièrement aux jours où tu offres des services de soins prénatals ou postnatals, sur une échelle de 1 à 5, 1 étant extrêmement insatisfait et 5 extrêmement satisfait, comment évalueriez-vous votre degré de satisfaction professionnelle actuelle?

(Thinking specifically about the days you provide ANC or PNC care, on a scale of 1-5, with 1 being extremely dissatisfied and 5 being extremely satisfied, how would you rate your current job satisfaction?)

◯ 1 Extrêmement insatisfait/Extremely dissatisfied
◯ 2 Insatisfait/Dissatisfied
◯ 3 Neutre/Neutral
◯ 4 Satisfait/Satisfied
◯ 5 Extrêmement satisfait/Extremely satisfied

Motivation du prestataire/Provider motivation

En réfléchissant plus particulièrement à votre travail dans son ensemble, y compris toutes vos tâches, peux-tu dire que tu es Fortement en accord, en Accord, ni en Accord ni en Désaccord, en Désaccord ou Fortement en désaccord avec chacune de ces affirmations?
12. Je me sens motivé à travailler aussi dur que je peux

(I feel motivated to work as hard as I can)

◯ Fortement en accord/Strongly agree
◯ en Accord/Agree
◯ ni en Accord ni en Désaccord/Neither agree nor disagree
◯ en Désaccord/Disagree
◯ Fortement en désaccord/Strongly disagree

---

13. La seule raison pour laquelle je fais ce travail est d'être payée
(The only reason I do this job is to get paid)

◯ Fortement en accord/Strongly agree
◯ en Accord/Agree
◯ ni en Accord ni en Désaccord/Neither agree nor disagree
◯ en Désaccord/Disagree
◯ Fortement en désaccord/Strongly disagree

---

14. Je me sens vidée de ma force émotionnelle à la fin de chaque jour
(I feel emotionally drained at the end of every day)

◯ Fortement en accord/Strongly agree
◯ en Accord/Agree
◯ ni en Accord ni en Désaccord/Neither agree nor disagree
◯ en Désaccord/Disagree
◯ Fortement en désaccord/Strongly disagree

---

15. Parfois, quand je me lève le matin, je redoute d'avoir à faire face à un autre jour au travail
(Sometimes when I get up in the morning, I dread having to face another day at work)

◯ Fortement en accord/Strongly agree
◯ en Accord/Agree
◯ ni en Accord ni en Désaccord/Neither agree nor disagree
◯ en Désaccord/Disagree
◯ Fortement en désaccord/Strongly disagree

---

16. J'accomplis des tâches qui doivent être faites sans que l'on me le demande ou dit
(I do things that need doing without being asked or told)

◯ Fortement en accord/Strongly agree
◯ en Accord/Agree
◯ ni en Accord ni en Désaccord/Neither agree nor disagree
◯ en Désaccord/Disagree
◯ Fortement en désaccord/Strongly disagree

---

17. Ce n'est pas grave si j'arrive parfois tard au travail
(It is not a problem if I sometimes come late to work)

◯ Fortement en accord/Strongly agree
◯ en Accord/Agree
◯ ni en Accord ni en Désaccord/Neither agree nor disagree
◯ en Désaccord/Disagree
◯ Fortement en désaccord/Strongly disagree

18. Je me sens fort peu engagé envers l'établissement de santé pour lequel je travaille
(I feel very little commitment to the health facility where I work)

◯ Fortement en accord/Strongly agree
◯ en Accord/Agree
◯ ni en Accord ni en Désaccord/Neither agree nor disagree
◯ en Désaccord/Disagree
◯ Fortement en désaccord/Strongly disagree

Opinions du prestataire/Provider opinions

En réfléchissant à ton expérience en matière d'offre de soins aux femmes enceintes et aux nouvelles mères, peux-tu dire que tu es Fortement en accord, en Accord, ni en Accord ni en Désaccord, en Désaccord ou Fortement en désaccord avec chacune de ces affirmations?
19. Il faut apprendre aux femmes enceintes et aux nouvelles mères la façon de prendre soin d'elles-mêmes car elles ont du mal à comprendre les comportements sains

(Pregnant and new mothers must be told how to care for themselves because they do not understand healthy behaviors)

◯ Fortement en accord/Strongly agree
◯ en Accord/Agree
◯ ni en Accord ni en Désaccord/Neither agree nor disagree
◯ en Désaccord/Disagree
◯ Fortement en désaccord/Strongly disagree

20. Les femmes enceintes ont de bonnes idées sur la façon de se maintenir en bonne santé

(Pregnant mothers have good ideas about how to keep themselves healthy)

◯ Fortement en accord/Strongly agree
◯ en Accord/Agree
◯ ni en Accord ni en Désaccord/Neither agree nor disagree
◯ en Désaccord/Disagree
◯ Fortement en désaccord/Strongly disagree

21. J'aime poser aux patients de partager leurs opinions, leurs histoires ou leurs idées
(I like to ask patients about their opinions, stories, or ideas)

◯ Fortement en accord/Strongly agree
◯ en Accord/Agree
◯ ni en Accord ni en Désaccord/Neither agree nor disagree
◯ en Désaccord/Disagree
◯ Fortement en désaccord/Strongly disagree

En réfléchissant à tes propres préférences personnelles, peux-tu dire que tu es Fortement en accord, en Accord, ni en Accord ni en Désaccord, en Désaccord ou Fortement en désaccord avec chacune de ces affirmations?

22. J'ai le désir d'essayer de nouvelles façons de faire des choses plutôt que de toujours faire les choses de la même manière

(I would like to try new ways of doing things rather than always doing things the same way)

◯ Fortement en accord
◯ en Accord
◯ ni en Accord ni en Désaccord
◯ en Désaccord
◯ Fortement en désaccord

23. Chaque personne que je rencontre, je la considère comme mon égale, même si nous sommes très différents (Every person I meet is equal to me even if we are very different)

◯ Fortement en accord/Strongly agree
◯ en Accord/Agree
◯ ni en Accord ni en Désaccord/Neither agree nor disagree
◯ en Désaccord/Disagree
◯ Fortement en désaccord/Strongly disagree

24. Dans quel service de l'établissement de santé préfères-tu travailler? (Veuillez choisir votre réponse.)

(In which area of the health facility do you most prefer to work? (Please choose your top answer.))

◯ Soins prénatals/ANC
◯ Consultation postnatale/PNC
◯ Maternité, Travail et accouchement/Maternity, Labor & Delivery
◯ Planification familiale/Family Planning
◯ Vaccinations/Immunizations
◯ Surveillance de la croissance et Réhabilitation nutritionnelle thérapeutique/Growth Monitoring and Therapeutic nutritional rehabilitation
◯ Intervention chirurgicale mineure/Minor surgery
◯ Services de soins palliatifs/Palliative care
◯ Consultation prénuptiale/Pre-marital consultation
◯ VCT, PTME et dépistage à l'initiative du prestataire (PIT)/VCT, PMTCT and PIT
◯ Consultation primaire curative/Primary curative consultation
◯ Soins et traitement pour les personnes vivant avec le VIH/SIDA--Care and treatment for person living with HIV/AIDS
◯ Hospitalisation/Hospitalization
◯ Services de stockage et de fourniture de médicaments/Drug storage & Supply services
◯ Position de leadership (Planification, S & E, Gestion du personnel ...)/Leadership position (Planning, M&E, Personnel management ...)
◯ Autre (veuillez préciser)/Other (please specify)

24a. Autre service de l'établissement de santé dans lequel tu préfères travailler:

____
(Other area in the health facility where you most prefer to work (top choice):)

25. Quel est ton deuxième choix quant au service de l'établissement de santé dans lequel tu préfères travailler? (What is your second choice for an area of the health center in which you prefer to work?)

◯ Soins prénatals/ANC
◯ Consultation postnatale/PNC
◯ Maternité, Travail et accouchement/Maternity, Labor & Delivery
◯ Planification familiale/Family Planning
◯ Vaccinations/Immunizations
◯ Surveillance de la croissance et Réhabilitation nutritionnelle thérapeutique/Growth Monitoring and Therapeutic nutritional rehabilitation
◯ Intervention chirurgicale mineure/Minor surgery
◯ Services de soins palliatifs/Palliative care
◯ Consultation prénuptiale/Pre-marital consultation
◯ VCT, PTME et dépistage à l'initiative du prestataire (PIT)/VCT, PMTCT and PIT
◯ Consultation primaire curative/Primary curative consultation
◯ Soins et traitement pour les personnes vivant avec le VIH/SIDA--Care and treatment for person living with HIV/AIDS
◯ Hospitalisation/Hospitalization
◯ Services de stockage et de fourniture de médicaments/Drug storage & Supply services
◯ Position de leadership (Planification, S & E, Gestion du personnel ...)/Leadership position (Planning, M&E, Personnel management ...)
◯ Autre (veuillez préciser)/Other (please specify)

25a. Autre service de l'établissement de santé dans lequel tu préfères travailler (deuxième choix):

____
(Other area in the health facility where you prefer to work (second choice):)

26. Quel est ton troisième choix quant au service de l'établissement de santé dans lequel tu préfères travailler?
(What is your third choice for an area of the health facility in which you prefer to work?)

◯ Soins prénatals/ANC
◯ Consultation postnatale/PNC
◯ Maternité, Travail et accouchement/Maternity, Labor & Delivery
◯ Planification familiale/Family Planning
◯ Vaccinations/Immunizations
◯ Surveillance de la croissance et Réhabilitation nutritionnelle thérapeutique/Growth Monitoring and Therapeutic nutritional rehabilitation
◯ Intervention chirurgicale mineure/Minor surgery
◯ Services de soins palliatifs/Palliative care
◯ Consultation prénuptiale/Pre-marital consultation
◯ VCT, PTME et dépistage à l'initiative du prestataire (PIT)/VCT, PMTCT and PIT
◯ Consultation primaire curative/Primary curative consultation
◯ Soins et traitement pour les personnes vivant avec le VIH/SIDA--Care and treatment for person living with HIV/AIDS
◯ Hospitalisation/Hospitalization
◯ Services de stockage et de fourniture de médicaments/Drug storage & Supply services
◯ Position de leadership (Planification, S & E, Gestion du personnel ...)/Leadership position (Planning, M&E, Personnel management ...)
◯ Autre (veuillez préciser)/Other (please specify)

26a. Autre secteur de l'établissement de santé dans lequel tu préfères travailler (troisième choix):

____
(Other area in the health facility where you prefer to work (third choice):)

Échelle du stress ressenti/Perceived Stress Scale

Ces questions permettent d'identifier les émotions et les pensées que tu as affichées au cours du dernier mois.

27. Au cours du dernier mois, à quelle fréquence t'es-tu senti bouleversé en raison d'un événement imprévu?

(In the last month, how often have you been upset because of something that happened unexpectedly?)

◯ Jamais/Never
◯ Rarement/Almost never
◯ Parfois/Sometimes
◯ Très souvent/Fairly often
◯ Toujours/Very often

28. Au cours du dernier mois, à quelle fréquence as-tu senti que tu n'étais pas en mesure de maîtriser les éléments importants de ta vie?
(In the last month, how often have you felt that you were unable to control the important things in your life?)

◯ Jamais/Never
◯ Rarement/Almost never
◯ Parfois/Sometimes
◯ Très souvent/Fairly often
◯ Toujours/Very often

29. Au cours du dernier mois, à quelle fréquence t'es-tu senti nerveux et "stressé"?
(In the last month, how often have you felt nervous and "stressed"?)

◯ Jamais/Never
◯ Rarement/Almost never
◯ Parfois/Sometimes
◯ Très souvent/Fairly often
◯ Toujours/Very often

30. Au cours du dernier mois, à quelle fréquence t'es-tu senti confiant quant à ta capacité de gérer tes problèmes personnels ?

(In the last month, how often have you felt confident about your ability to handle your personal problems?)

◯ Jamais/Never
◯ Rarement/Almost never
◯ Parfois/Sometimes
◯ Très souvent/Fairly often
◯ Toujours/Very often

31. Au cours du dernier mois, à quelle fréquence t'es-tu senti que tu ne pouvais pas prendre en charge toutes les choses que tu devais faire ?

(In the last month, how often have you found that you could not cope with all the things that you had to do?)

◯ Jamais/Never
◯ Rarement/Almost never
◯ Parfois/Sometimes
◯ Très souvent/Fairly often
◯ Toujours/Very often

32. Au cours du dernier mois, à quelle fréquence t'es-tu senti que les choses allaient comme tu le voulais?

(In the last month, how often have you felt that things were going your way?)

◯ Jamais/Never
◯ Rarement/Almost never
◯ Parfois/Sometimes
◯ Très souvent/Fairly often
◯ Toujours/Very often

33. Au cours du dernier mois, à quelle fréquence as-tu pu être en mesure de contrôler les irritations dans ta vie?

(In the last month, how often have you been able to control irritations in your life?)

◯ Jamais/Never
◯ Rarement/Almost never
◯ Parfois/Sometimes
◯ Très souvent/Fairly often
◯ Toujours/Very often

34. Au cours du dernier mois, à quelle fréquence as-tu senti que tu prenais le dessus sur tout ?

(In the last month, how often have you felt that you were on top of things?)

◯ Jamais/Never
◯ Rarement/Almost never
◯ Parfois/Sometimes
◯ Très souvent/Fairly often
◯ Toujours/Very often

35. Au cours du dernier mois, à quelle fréquence t'es-tu mis en colère en raison des choses qui échappaient à ton contrôle?

(In the last month, how often have you been angered because of things that were outside of your control?)

◯ Jamais/Never
◯ Rarement/Almost never
◯ Parfois/Sometimes
◯ Très souvent/Fairly often
◯ Toujours/Very often

36. Au cours du dernier mois, combien de fois as-tu senti que les difficultés prenaient tellement de place que tu ne pourrais pas toutes les surmonter ?

(In the last month, how often have you felt difficulties were piling up so high that you could not overcome them?)

◯ Jamais/Never
◯ Rarement/Almost never
◯ Parfois/Sometimes
◯ Très souvent/Fairly often
◯ Toujours/Very often
