# Supplementary material for: Nurses’ and midwives’ experiences of providing group antenatal and postnatal care at 18 health centers in Rwanda: A mixed methods study
Source: PLoS One. 2019 Jul 11;14(7):e0219471. doi: 10.1371/journal.pone.0219471 (PMC6622527; doi:10.1371/journal.pone.0219471)
Supplement: S2 Doc — (PDF) [file pone.0219471.s002.pdf]

Appendix 4: PTBi Rwanda Group Antenatal/Postnatal Care Providers Focus Group Guide

A. Welcome note

Welcome and thank you for volunteering to take part in this focus group. You have been asked to participate as your point of view is important to us. We understand to you are busy and appreciate your time.

B. Introduction of the topic

This focus group discussion (FGD) is designed to understand the experiences of providers who have been group antenatal or postnatal care facilitators. The FGD will take no more than two hours. We intend to audio-record our discussion to ensure we capture your thoughts accurately. If everyone agrees, we will start the audio recording now.

(If yes, switch on the recorder).

C. Anonymity

Despite being recorded, I would like to assure you that the discussion is confidential and the audio recording will not include any information to identify participants. The audio recordings will be kept in an encrypted file on a computer. The transcribed notes of the focus group will contain no personal identifiable information. You should try to answer and comment as accurately and truthfully as possible. I and the other focus group participants would appreciate it if you would refrain from discussing the comments of other group members outside the focus group. If there are any questions or discussions that you do not wish to answer or participate in, you do not have to do so; however please try to answer and be as involved as possible.

D. Ground rules/ Guidelines

- The most important rule is that only one person speaks at a time. There may be a temptation

to jump in when someone is talking but please wait until they have finished.

- There are no right or wrong answers, only differing points of view
- You do not have to speak in any particular order
- When you do have something to say, please do so. There are many of you in the group and it is important that I obtain the views of each of you
- You do not need to agree with others, but you must listen respectfully as others share their views
- Rules for cellular phones if applicable. For example: We ask that you turn off your phones. If you cannot and if you must respond to a call, please step out of the room to do so and rejoin us as quickly as you can.

Does anyone have any questions?

OK, let's begin. Participant/Moderator intro: Please share with the group your name and where you work (moderator to go first as an example).

E. Clarification

When we talk about group antenatal and postnatal care today, we are referring to the program called "Ibaruke Neza Mubyeyi," in which a group of about 10 pregnant women meet together 3 times during pregnancy and once after birth, together with a provider and a community health worker.

F. Questions

Introduce the objective of these questions. For example, we are interested in learning about your experiences as Ibaruke Neza Mubyeyi facilitators.

Transitioning questions

All the members of this group have been Ibaruke Neza Mubyeyi facilitators. Please share with the group:

- What is the most interesting story you remember from something that happened at one of the group care visits?

Activity 1: Get to know participants' initial reaction to the invitation to GANC/GPNC

- What did you think about group care when you first heard of it?

Activity 2: Effect on work

- How did group care affect your work activities?

Activity 3: Keys to success

- What do you think are the reasons that Ibaruke Neza Mubyeyi was successful or had difficulties at your health center?

Activity 4: Effects on mothers

- How do you think Ibaruke Neza Mubyeyi affected mothers who attended the sessions?

Activity 5: Changes or improvements

- What changes would you make to Ibaruke Neza Mubyeyi to make it better?

G. Conclusion

Thank you for participating. This has been a very successful discussion. Your opinions are valuable. We hope you have found the discussion interesting. If there is anything you are unhappy with or wish to complain about, please contact the Principal Investigator or speak to me later. I would like to remind you that any comments and feedback are confidential and anything you share will help improve ANC and PNC services in Rwanda.
